# Estimates of Current Capacity for Diagnosing Alzheimer’s Disease in Sweden and the Need to Expand Specialist Numbers

**DOI:** 10.14283/jpad.2023.94

**Published:** 2023-09-15

**Authors:** Soeren Mattke, A. Gustavsson, L. Jacobs, S. Kern, S. Palmqvist, M. Eriksdotter, I. Skoog, B. Winblad, A. Wimo, L. Jönsson

**Affiliations:** 1 University of Southern California, 635 Downey Way, #505N, Los Angeles, California 90089 USA; 2 Quantify Research, Stockholm, Sweden; 3 Div of Neurogeriatrics, Dept of Neurobiology, Care Sciences and Society, Karolinska Institutet, Solna, Sweden; 4 Neuropsychiatric Epidemiology Unit, Sahlgrenska Academy, University of Gothenburg, Gothenburg, Sweden; 5 Department of Psychiatry Cognition and Old Age Psychiatry, Sahlgrenska University Hospital, Mölndal, Sweden; 6 Clinical Memory Research Unit, Department of Clinical Sciences, Lund University, Malmö, Sweden; 7 Memory Clinic, Skåne University Hospital, Malmö, Sweden; 8 Div of Clinical Geriatrics, Dept of Neurobiology, Care Sciences and Society, Karolinska Institutet, Huddinge, Sweden; 9 Theme Inflammation and Aging, Karolinska university hospital, Huddinge, Sweden

**Keywords:** Disease-modifying treatment, preparedness, capacity, Alzheimer’s disease, Sweden

## Abstract

**Background:**

The emergence of disease-modifying Alzheimer’s (AD) treatments provides new hope to patients and families but concerns have been raised about the preparedness of healthcare systems to provide timely access to such treatments because of a combination of a complex diagnostic process and a large prevalent pool.

**Objectives:**

We assess the preparedness of Sweden, a high-income country known for its dementia-friendly policies, to diagnose AD patients eligible for treatment within a six-month window, given current capacity for specialist evaluations and biomarker testing. We calculate the investment requirements for Sweden to achieve this target over a timeframe of 20 years.

**Design:**

Desk research to identify data for population, mortality, disease burden, cost of services and current capacity, expert consultation to inform assumptions about patient journey, and use of a Markov model to predict waiting times. The model simulates the patients’ journey through different evaluation stages: initial evaluation by a primary care specialist, neurocognitive testing by an AD specialist, and confirmatory biomarker testing with PET scanning or cerebrospinal fluid (CSF) testing. The model assumes specialist appointments and PET scans are capacity constrained, and patients progress from cognitively normal to MCI and from MCI to dementia in the resulting waiting times.

**Measurements:**

Projected waiting times for diagnosis of eligibility for disease-modifying Alzheimer’s treatment from 2023 to 2042 assuming current capacity, assuming 20% of Swedish residents aged 60 years and above would seek an evaluation for cognitive decline. Investments required to scale capacity up to reach target of providing diagnosis within six months on average.

**Results:**

Initial average waiting times for AD specialist appointments would be around 21 months in 2023 and remain around 55 months through 2042, as demand would continue to outstrip supply throughout the 20-year model horizon. Waiting times for biomarker testing would be stable at less than four weeks, as patients would be held up in the queue for their first specialist consultations, and use of CSF testing is widely accepted in Sweden. An additional 25% of AD specialists would have to be added above the current growth trend to reduce waiting times to less than 6 months at an average annual cost of approximately 805 million SEK. The increased cost of volume of biomarker testing would amount to about 106 million SEK per year.

**Conclusions:**

At current capacity, the Swedish healthcare system is unable to provide timely diagnosis of patients eligible for disease-modifying AD treatment. Although future diagnostic technologies, such as digital cognitive assessments and blood tests for the AD pathology, might decrease demand for capacity-constrained services, substantial investments will be required to meet a target of less than six months of waiting time for a diagnosis.

## Introduction

**R**ecent advancements in Alzheimer’s disease (AD) drug development (1–3, 4) offer hope for a first generation of disease modifying treatments (DMT) to become available to patients soon. Two monoclonal antibodies directed against amyloid, aducanumab and lecanemab, have received regulatory approval in the United States (U.S.) under the Food and Drug Administration (FDA) accelerated approval provisions (5, 6). Lecanemab is under review by the European Medicines Agency (EMA) (7). After drugs receive regulatory approval, payers need to decide whether to fund them; often this is contingent on the assessment of their expected clinical benefits weighed against potential side-effects and costs (8).

While lecanemab is currently undergoing review by the EMA, European policy makers and healthcare providers have the opportunity to focus on preparing for an expectedly significant paradigm shift in the management of patients with AD. In the absence of effective DMTs today, this management has focused on symptomatic relief and supportive care (9), in addition to non-pharmacological disease prevention strategies which have shown to reduce risks of developing dementia (10, 11). The majority of persons with dementia are only diagnosed in the late stages of their disease, too late for meaningful disease-modifying intervention. This has been suggested to be because of reasons including stigma, false perceptions of normal aging and a perceived lack of treatment options (12). The perceived benefits of an early diagnosis may be weighed against its risks (13), deferring some from seeking care for their symptoms or causing physicians to be reluctant to prioritize dementia detection and diagnosis. However, this is expected to shift if an effective DMT becomes approved and is made available, resulting in increased pressure on all parts of the AD diagnostic workup as well as subsequent treatment and monitoring of patients.

Healthcare systems across the globe are ill-prepared for this new paradigm (14, 15). Previously studied healthcare systems generally lack resources and training to effectively diagnose more than small portions of the numerous persons with AD (16). To change this, policy makers need information on what resources are available today, and what investments are needed to prepare for an increased demand for timely and accurate diagnosis of AD.

In this study, we chose to focus on Sweden, a high-income country with universal access to a national healthcare system that is primarily funded by taxes. Sweden ranks amongst the highest in Europe in terms of dementia-friendly policies (17), but there are also reported challenges. These include the lack of dementia experts (herein referred to as AD specialist) and geriatric competence across all levels of healthcare staff (18, 19), misdiagnosis and delays in diagnosis (20). Another underlying complexity is that healthcare is organized in 21 regions with considerable independence and self-determination regarding healthcare, leading to variability in care service provision. These challenges will be exacerbated should DMTs be made available to patients. Our objective was to assess the currently available resources for diagnosing patients with AD for potential eligibility to a DMT in Sweden, project waiting times for the diagnostic pathway under current resources and estimate the investment required to reduce waiting times to less than six months on average. The six months is aligned with Swedish law, in which a ‘care guarantee’ says patients should be assessed at a specialist visit within three months of referral and start treatment within three months of a treatment decision (9 kap 1 §, HSL; 2 kap 3–4 §§, PL). We limited our study to the availability of AD specialists and biomarker testing, while acknowledging that other resources including more primary care specialists, neuropsychologists, and magnetic resonance imaging (MRI) capacity are expected to be needed as well.

## Methods

### Model description

We used a Markov model that has been described in detail in earlier publications (21, 22). In short, it simulates the journey of patients seeking evaluation for subjective memory complaints or as part of a wellness exam in primary care with two interacting layers. The first layer captures one of four true health states: cognitively normal, mild cognitive impairment (MCI) due to AD, MCI due to other causes, and dementia using age and sex-specific estimates for incidence and prevalence of MCI and dementia. The second layer captures a patient’s journey through different evaluation stages: initial evaluation by a primary care specialist, neurocognitive testing by an AD specialist, and confirmatory biomarker testing with positron emission tomography (PET) scanning or cerebrospinal fluid (CSF) testing. The model assumes specialist appointments and PET scans are capacity-constrained, and patients progress from cognitively normal to MCI and from MCI to dementia in the resulting waiting times.

### Patient journey

We consider a hypothetical scenario where a DMT becomes available in Sweden in 2023. This is expected to imply changes to current clinical practice, including patient behaviors, the patient pathway, and activities from first contact with health care to diagnosis and treatment decision and the allocation of time of specialists on this particular patient group. At present, we cannot know the extent of these changes and we therefore need to rely on assumptions. We use the following assumptions, guided by local expert input, on how patients progress through the different steps of their journey in our base case scenario:

- Starting in 2023, 20% of all individuals of age 60 and above, who have never been evaluated for cognitive decline, undergo a brief cognitive test, like the minimental state examination (MMSE) or Montreal Cognitive Assessment (MoCA), in primary care either because of a subjective memory complaint or as part of a routine assessment. Access to these visits is assumed to be unconstrained. Ten percent of individuals, who were previously tested negative at any stage of their journey, return for a repeated evaluation each year.
  - ○ As alternative scenarios, we assume initial uptake to be 10% and 40%.
- Of the individuals found to be cognitively impaired, 80% with suspected MCI based on the brief cognitive assessment are referred to an AD specialist, while the remaining 20% are diagnosed with manifest dementia or cognitive impairment of reversible etiology, such as depression or alcohol use, and treated in primary care settings.
- During the first visit, the AD specialist performs neurocognitive testing and orders biomarker testing for 85% of patients with confirmed MCI, having identified a different etiology in 15%.
  - ○ According to Swedish experts, 90% of patients with MCI performing biomarker testing would undergo a lumbar puncture for CSF testing, which is assumed not to be capacity-constrained, to establish the Alzheimer’s pathology. We assume that the remaining 10% of cases would require a PET scan.
- All patients return for a second specialist visit to discuss all findings and decide on a care plan. The simulation prioritizes second specialist visits over first, i.e., slots for first visits are only made available if no patient waits for his or her second visit.

At each step, patients can be found not to have MCI due to AD based on test results and exit the queue for that year. We model waiting times over a 20-year horizon from 2023 to 2042. For each of the three scenarios, we estimated the investment requirements to reduce average waiting times to less than six months.

### Data

#### Population size, mortality, and disease prevalence

The model was populated with national estimates on the size of the Swedish population above 60 years of age, from year 2020 and projections until 2050, stratified by sex and 5-year age category (23). National mortality rates were available for the same categories (24). Prevalence estimates for Aβ-positive MCI due to AD and AD dementia were sourced from a recent review, assuming western European estimates are applicable to Sweden (25). Separate prevalence estimates for AD dementia were available for sex and age categories, whereas for MCI due to AD, they were assumed to be the same for males and females. Prevalence data on MCI due to AD were not available for age groups below 60 years, for which assumptions based on extrapolation were used in absence of better data (0.9% for men and women aged 50–54, and 1.5% aged 55–59).

### Resource availability

The number of PET scanners in Sweden was estimated based on separate surveys suggesting an increase from three scanners in 2005 to 23 in 2019 (26, 27). We extrapolated on these data assuming a linear increase based on the historic trend. It was assumed that each scanner has a capacity of 2000 scans per year. The clinical specialists, who are involved in the formal diagnosis of patients with dementia in Sweden, include primarily geriatricians, psychiatrists, and neurologists, with large variation across regions. National statistics on the number of such specialists were extracted from the National Board of Health and Welfare (28). However, only a subset of these is expected to have adequate competence to make an AD diagnosis (herein referred to as AD specialists), because a part of these professionals focusses on other patient groups. A panel of Swedish clinical experts was asked about the proportion of each professional with such competence, resulting in an average estimate of 85%, 25% and 50% of the projected year 2023 representing 644 geriatricians, 2,321 psychiatrists and 576 neurologists, respectively. Again, we used a linear projection to estimate future numbers of specialists, based on the historical trend. We assume that specialists can offer 1,130 consultations per year (on average 5 consultations per work day) and that existing specialists will devote on average 5% of these consultations to evaluation of eligibility for a disease-modifying AD treatment.

### Costs

The costs of specialist visits and biomarker testing in the diagnostic workup of AD and dementia were retrieved from administrative reports and published literature and inflated to 2023 values. Costs are reported in Swedish crowns (SEK) but can be converted to EUR or USD at about 10 SEK/EUR or USD. The cost of a PET scan was estimated to be 17,891 SEK (29) and that of CSF testing 8,433 SEK (5,051 SEK for the lumbar puncture (30) and 3,293 SEK for the CSF analysis of Aβ42/Aβ40, total tau and phosphorylated tau (31)). The salary cost of an AD specialist, including payroll and overhead was estimated at 2.05 million SEK per year (32). Future costs were inflated by 3% annually.

## Results

### Estimated waiting times under status quo assumptions

**Figure 1:**
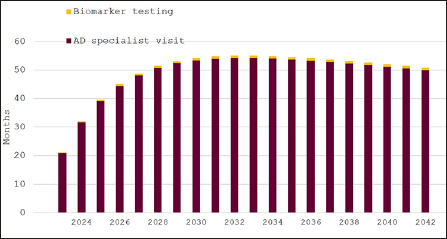
Expected waiting times for diagnosis of eligibility for disease-modifying Alzheimer’s disease treatment in Sweden, 2023–2042, 20% participation in cognitive screening

Figure 1 displays the waiting times in our base case scenario, which assumes that 20% of Swedish residents aged ≥60 years (around 560,000 people in 2023) would seek an evaluation for cognitive decline. Initial average waiting times for AD specialist appointments would be around 21 months in 2023 and remain around 55 months through 2042, as demand would continue to outstrip supply throughout the 20-year model horizon. Waiting times for biomarker testing would be stable at less than four weeks, as patients would be held up in the queue for their first specialist consultations.

In 2023, there would be 79,987 AD specialist visits at a cost of around 130 million SEK (∼13 million EUR/USD) and 19,582 confirmatory biomarker tests at a cost of approximately 182 million SEK (∼18 million EUR/USD) with an uptake of cognitive screening of 20%. (Table 1) The number and cost of visits and biomarker tests does not change with uptake of screening because of saturated specialist capacity.

**Table 1:** Estimated spending given current infrastructure – Year 2023

	**Base case 20% uptake of screening**		**40% uptake of screening**		**10% uptake of screening**	
	**Number**	**Cost (SEK)**	**Number**	**Cost (SEK)**	**Number**	**Cost (SEK)**
AD specialist visits	79,987	130,244,821	79,987	130,244,821	79,987	130,244,821
CSF tests	17,624	147,051,944	17,624	147,051,944	17,624	147,051,944
PET scans	1,958	35,033,978	1,958	35,033,978	1,958	35,033,978

Note: 10 SEK is approximately 1 EUR or USD at the 2023 exchange rate; AD: Alzheimer’s disease; CSF: Cerebrospinal fluid; PET: Positron emission tomography; SEK: Swedish crowns

Over time, the average annual number of specialist visits are expected to increase, while fewer biomarker tests are conducted because the model prioritizes second visits over first visits, i.e., the waiting list for visits prior to biomarker testing grows disproportionately. On average over the 20-year horizon, there would be 90,855 AD specialist visits at a cost of around 201 million SEK (∼20 million EUR/USD) and 15,524 confirmatory biomarker tests at a cost of approximately 229 million SEK (∼23 million EUR/USD) on average per year with 20% uptake. (Table 2)

**Table 2:** Estimated annual spending given current infrastructure over 20-year horizon

	**Base case 20% uptake of screening**		**40% uptake of screening**		**10% uptake of screening**	
	**Number**	**Cost (SEK)**	**Number**	**Cost (SEK)**	**Number**	**Cost (SEK)**
AD specialist visits	90,855	201,207,817	90,855	201,207,817	90,855	201,207,817
CSF tests	13,971	191,853,708	12,772	157,597,347	13,520	141,295,901
PET scans	1,552	37,250,367	672	16,070,381	712	33,662,645

Note: 10 SEK is approximately 1 EUR or USD at the 2023 exchange rate; AD: Alzheimer’s disease; CSF: Cerebrospinal fluid; PET: Positron emission tomography; SEK: Swedish crowns

**Figure 2:**
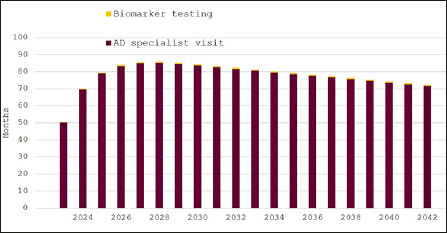
Expected waiting times for diagnosis of eligibility for disease-modifying Alzheimer’s disease treatment in Sweden, 2023–2042, 40% participation in cognitive screening

Figure 2 depicts the effect of increasing the uptake of evaluation for cognitive decline to 40% of Swedish residents. Waiting times for specialist consultations would be initially 50 months in 2023, peak at 85 months in 2028 and remain over 70 months until 2042. Waiting times for biomarker testing would remain less than four weeks throughout the model horizon. Doubling the number of individuals seeking an evaluation would not change the number of specialist visits, because supply is already fully saturated under the previous assumption of 20% uptake. As a consequence of the constrained specialist capacity, the number of biomarker tests also remains almost unchanged (Table 2)

**Figure 3:**
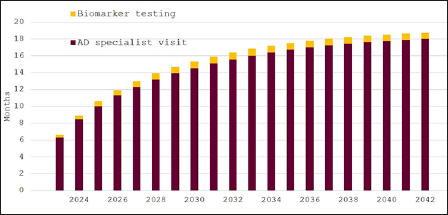
Expected waiting times for diagnosis of eligibility for disease-modifying Alzheimer’s disease treatment in Sweden, 2023–2042, 10% participation in cognitive screening

Our last scenario assumes that only 10% of Swedish residents (aged 60 years and older) would seek cognitive evaluation each year and Figure 3 shows the resulting waiting times. Estimated waiting times for specialist appointments would increase continuously from around six months in 2023 to 18 months in 2042. Waiting times for biomarker testing would remain below one month. The average number of specialist visits would again remain constant, whereas the number of confirmatory biomarker tests would fall to an average of 14,232 at a cost of around 175 million SEK (∼17 million EUR/USD) per year.

### Projected investment requirements

The waiting times projections under different assumptions for care-seeking behavior reveal that a lack of specialists would be the rate limiting factor in Sweden, whereas biomarker testing capacity appears sufficient. We estimated the cost of adding AD specialists on top of the projected growth trend to reach our target wait time of no more than six months and calculated the ensuing additional cost for biomarker testing. We assumed that the additional specialists would be fully devoted to evaluation of potential DMT candidates.

We estimated that 24%, 25%, and 45%, respectively, of AD specialists would have to be added above the current growth trend to reduce waiting times to less than 6 months in our scenarios with 10%, 20% and 40% uptake of cognitive screening. This relative increase translates into 340, 354 and 637 added full time specialists, respectively, in 2023 with numbers increasing to 432, 450 and 810 in 2042.

In 2023, and assuming 20% uptake of screening, those specialists would provide around 320,000 additional visits at a cost of 521 million SEK (∼52 million EUR/USD), as their time is exclusively devoted to evaluation of treatment eligibility, and there would be approximately 30,500 additional biomarker tests at a cost of 284 million SEK (∼28 million EUR/USD) (Table 3).

**Table 3:** Cost and added service volume to reduce average annual waiting times to below six months, – Year 2023

	**Added services because of investment 20% uptake of screening**		**40% uptake of screening**		**10% uptake of screening**	
	**Number**	**Cost (SEK)**	**Number**	**Cost (SEK)**	**Number**	**Cost (SEK)**
AD specialist visits	319,949	520,979,284	639,898	1,041,958,567	303,952	494,930,319
CSF tests	27,444	228,994,883	53,771	481,762,868	27,444	228,994,883
PET scans	3,049	54,556,243	5,975	35,928,758	3,049	54,556,243

Note: 10 SEK is approximately 1 EUR or USD at the 2023 exchange rate; AD: Alzheimer’s disease; CSF: Cerebrospinal fluid; PET: Positron emission tomography; SEK: Swedish crowns

Over the 20-year time horizon, the cumulative effect of adding specialists would lead to on average 636,422 additional visits at a cost of approximately 805 million SEK (∼81 million EUR/USD) per year. Average volume of biomarker testing would increase to 8,103 tests at an added cost of about 106 million SEK (∼11 million EUR/USD). Adding 45% more AD specialists to reduce waiting times to below six months with 40% uptake would require an average annual investment of 1,610 million SEK (∼161 million EUR/USD) to generate 726,843 additional visits. Lastly, around 345,251 visits at a cost of 765 million SEK (∼77 million EUR/USD) would have to be added on average per year to keep waiting times below six months if uptake of screening were 10% (Table 4).

**Table 4:** Cost and added service volume to reduce average annual waiting times to below six months, average annual change over 20-year horizon

	**Added services because of investment 20% uptake of screening**		**40% uptake of screening**		**10% uptake of screening**	
	**Number**	**Cost (SEK)**	**Number**	**Cost (SEK)**	**Number**	**Cost (SEK)**
AD specialist visits	363,422	804,831,270	726,843	1,609,662,540	345,251	764,589,706
CSF tests	7,293	88,589,390	12,236	133,957,383	8,925	143,626,708
PET scans	810	17,569,754	644	13,855,282	470	21,157,476

Note: 10 SEK is approximately 1 EUR or USD at the 2023 exchange rate; AD: Alzheimer’s disease; CSF: Cerebrospinal fluid; PET: Positron emission tomography; SEK: Swedish crowns

**Figure 4:**
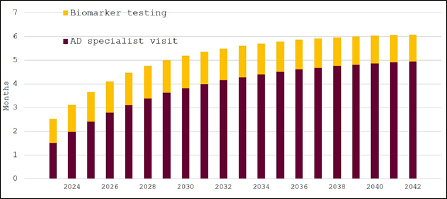
Expected waiting times for diagnosis of eligibility for disease-modifying Alzheimer’s disease treatment in Sweden, 2023–2042, 20% participation in cognitive screening, 25% expansion of specialist capacity

Figure 4 shows the projected waiting time for our base case scenario with expansion of specialist capacity. Waiting times for specialist visits would remain below six months and for biomarker testing at around four to six weeks.

## Discussion

We have analyzed waiting times in the diagnostic pathway to determine eligibility for a disease-modifying AD treatment in Sweden and found that projected waiting times are expected to exceed 21 months in 2023 and increase to around 50 months by 2028 and remain on that level for the course of the model horizon. This projection is comparable to those for the U.S. (22) and England (33) with around 50 months for both countries, and similar to those countries a shortage of AD specialists causes most of the waiting times. This result is counterintuitive for two reasons. First, the included age range in the U.S. and England started at age 50, whereas the Swedish experts advised us to restrict to age 60 and older. This change has a substantial impact on demand because the age cohort of 50 to 60 years is quite numerous, and, while its disease burden is low, many false positives would be referred to a specialist evaluation. Second, Sweden has a comparatively high number of AD specialists per capita, 13.6 AD specialists per 100,000 population, compared to the G7 average of 11 and well above the estimates for the U.S. and England with 8.8 and 5.0, respectively. The long waiting times for specialist appointments is explained by the comparatively low visit volume for specialists in Sweden, which experts estimated at 1,130 per year compared to 1,776 in England, 2,860 in the U.S. (22), and 3,744 in China (35), because Swedish AD specialists have many responsibilities in addition to patient care, such as administrative tasks, training of residents and research.

In contrast, Sweden relies heavily on CSF analysis for 90% of expected biomarker tests, which is more scalable than PET scanning, whereas in other countries there is greater reluctance to undergo lumbar punctures: the corresponding estimates are 10% for the U.S. (22), 42% for England (34) and 63% for China (35).

As a result, the investment requirements into AD specialists to achieve the target of waiting times below six months with around 800 million SEK would be almost an order of magnitude larger than that into additional capacity for biomarker testing with around 106 million SEK per year over the model horizon. To put this amount into perspective, our estimated investment of approximately 800 million SEK (∼80 million EUR/ USD) in 2023 under the base case scenario corresponds to approximately 0.13% of annual healthcare spending or 76 SEK per capita in Sweden that year.

We caution, however, that adding highly-trained specialists is not as straightforward as installing imaging equipment and it may not be possible to train and/or recruit those specialists in the short term. It takes five years of residency to become a geriatrician, neurologist, psychiatrist and after this additional clinical training in memory clinics for 1–2 years to become proficient at diagnosing/treating cognitive disorders. Conversely, improvements in diagnostic technology could lessen the need to expand specialist capacity. Digital cognitive tests (36, 37) could streamline neurocognitive evaluations and facilitate task-shifting to lesser-trained clinicians. In addition, there are several scalable blood-based biomarkers to determine Alzheimer’s pathology in advanced stages of development (38, 39), which could reduce the need for lumbar punctures, thereby freeing up specialist clinics.

### Limitations

This analysis has several limitations. We used a combination of published data and expert input to estimate waiting times and cost. Many of the inputs are uncertain, including prevalence estimates for MCI which are not based on local studies but on global meta-analyses, because these meta-analyses have not found sufficient evidence of cross-country differences. Actual costs might differ from our estimates in either direction and vary substantially. Lower than expected demand of patients to be evaluated for an AD treatment might reduce the need to expand diagnostic infrastructure, which the scenario with 10% uptake of cognitive screening illustrates. While, as we mentioned, progress in diagnostic technology, such as blood tests for the AD pathology and digital cognitive tests, could become substitutes for existing technologies and reduce demand, we caution that those technologies may not be approved or fully implemented in time for use in routine clinical practice, especially in primary care, prior to the launch of an initial disease-modifying AD treatment. Our model horizon of 20 years does not take future technological changes into account which make long-term outcomes highly uncertain. Our estimates did not consider constraints on primary care capacity, which is likely to become another bottleneck. Neither did the model consider other health care staff (e.g., neuropsychologists and occupational therapists) who are involved in the diagnostic process, nor MRI capacity which may be a bottleneck already before treatment initiation to rule out patients with potential contraindications. Lastly, we only analyzed capacity and demand for the diagnostic phase; capacity for treatment delivery with infusions and MRI and clinical monitoring might be constrained as well. The specific nature of such treatment would dictate the additional investment requirements into delivery and monitoring capacity, which could be substantial in the case of the amyloid-targeting antibodies.

## Conclusion

As in other studied countries, there is a need for substantial investments to enable the timely identification of patients eligible for DMTs in Sweden. Specialists with the competence and capacity to assess and diagnose AD patients are lacking, and biomarker capacity require scaling up. Without substantial investments in both categories, waiting times will quickly exceed four years on average, precluding many from the opportunity to potentially delay their disease with a future DMT.

## Notes

*Ethical standards:* No ethics approval was required for this study because it does not involve any personal data but was instead based on national statistics, published estimates and expert assumptions.
